# A randomized, controlled field study to assess the efficacy and safety of lotilaner (Credelio™) in controlling ticks in client-owned cats in Europe

**DOI:** 10.1186/s13071-018-2967-5

**Published:** 2018-07-13

**Authors:** Daniela Cavalleri, Martin Murphy, Wolfgang Seewald, Steve Nanchen

**Affiliations:** Elanco Animal Health, Mattenstrasse 24a, CH-4058 Basel, Switzerland

**Keywords:** Credelio, Lotilaner, Fipronil, Cat, Tick, Efficacy, Field study, Safety, Europe

## Abstract

**Background:**

There is a continuing need for novel approaches to tick infestations treatment and control in cats. Lotilaner, an isoxazoline with rapid onset of action, has proven its efficacy against ticks in laboratory studies. A study was undertaken to confirm lotilaner’s efficacy and safety in client-owned cats, at the minimum dose of 6.0 mg/kg, against the most common ticks infesting cats in Europe.

**Methods:**

Twenty clinics in Germany, Hungary and Portugal participated in the study. Households with no more than three cats were randomized 2:1 to a lotilaner or fipronil group. The first household cat with at least three live, attached ticks was the primary cat. Treatments were dispensed on days 0, 28 and 56 for owner administration. Tick counts were performed on days 0, 7, 14, 21, 28, 42, 56, 70 and 84 (primary cats) and supplementary cats were assessed for safety only, on days 28, 56 and 84. Efficacy was assessed by comparing mean day 0 live attached tick counts with subsequent counts.

**Results:**

Most frequently retrieved ticks were *Ixodes ricinus*, *Rhipicephalus sanguineus* and *Dermacentor reticulatus*, with *Ixodes hexagonus* also present. In the lotilaner group (*n* = 112) efficacy (based on geometric mean tick counts) was between 98.3–100%. For fipronil (*n* = 57), efficacy was between 89.6–99.6%, with live attached ticks present on some cats at all time points. Mean tick counts in lotilaner-treated cats were significantly lower than in fipronil-treated cats on days 21, 28, 42 and 56 (*P* < 0.05). The mean percent efficacy over all post-enrolment visits was 99.6% and 96.4% (lotilaner and fipronil group, respectively), (*P* < 0.0001). Lotilaner was superior to fipronil for efficacy averaged over all time points (*P* < 0.0001) and on individual assessment days (day 14 to 70, *P* < 0.0394); it was non-inferior to fipronil on the other days. Owners successfully administered all treatments, and both products were well tolerated.

**Conclusions:**

Credelio^TM^ was effective and safe for the treatment of tick infestations in client-owned cats. Efficacy lasted one month and lotilaner was superior to fipronil on most assessment days. Cure rates ranged between 94.5–100% for lotilaner and 68.4–98.2% for fipronil.

**Electronic supplementary material:**

The online version of this article (10.1186/s13071-018-2967-5) contains supplementary material, which is available to authorized users.

## Background

Favourable epidemiological conditions due to climate change and changing human behaviours leading to increased risk of tick exposure for humans and pets, have been linked to a widening geographical spread and increasing abundance of the ixodid ticks, *Ixodes ricinus*, *Dermacentor reticulatus* and *Rhipicephalus sanguineus* [1–4]. This increases the risk for vector-borne diseases such as tick-borne encephalitis, babesiosis, anaplasmosis and ehrlichiosis.

Topical tick control products have been the mainstay of treatment. However, there are now reports of resistance or tolerance to common topically applied acaricides [5]. This highlights the need for novel approaches to the treatment of tick infestations in pets. The emergence of the isoxazolines has begun to address that need. Acting with a unique mode of action, these molecules have been shown to be effective in treating ectoparasitic infestations on dogs [6–8].

Amongst the available isoxazolines, afoxolaner, fluralaner, sarolaner and lotilaner are approved as oral formulations for the control of tick and flea infestations in dogs. Lotilaner (Credelio™, Elanco) is the newest member of this class and is already available as a flavoured chewable tablet formulation for dogs [9]. Laboratory studies in dogs demonstrated that lotilaner quickly begins killing induced infestations with *I. ricinus*, and has sustained activity against new infestations with *Ixodes scapularis*, *I. ricinus*, *Dermacentor variabilis*, *D. reticulatus* and *Rhipicephalus sanguineus* for at least 35 days [10, 11].

The only isoxazolines approved for use in cats are fluralaner (Bravecto®; Merck Animal Health, Madison, NJ, USA) [12] and sarolaner (Stronghold Plus®, Zoetis SA, Louvain-la-Neuve, Belgium) [13], both as solutions for topical application. A flavoured oral formulation would fulfil the need for a rapidly effective, orally administered ectoparasiticide with sustained activity for the control of ticks and fleas in cats and kittens.

The safety and efficacy of lotilaner tablets against ticks and fleas in cats were assessed in a number of pilot (unpublished data) and pivotal [14] studies. A pivotal tolerance study in 8-week-old kittens has shown lotilaner to be safe at doses up to 130 mg lotilaner/kg (five times the maximum therapeutic dose, with a dose banding of four) for monthly administration over 8 months. Efficacy of the final formulation was demonstrated following oral administration at a minimum dose rate of 6.0 mg/kg against fleas (*Ctenocephalides felis*) and *I. ricinus* for 1 month in pivotal field and laboratory studies [15–17].

In this randomised, assessor-blinded, multicentre trial, the authors evaluated the efficacy, safety, and palatability of lotilaner (Credelio^TM^ chewable tablets for cats) administered at a minimum dose rate of 6.0 mg/kg to client-owned cats naturally infested with ticks, under field conditions in Europe. Eliminall® Spot-on (fipronil 50 mg, KRKA d.d. Novo mesto, Novo Mesto Slovenia) was used as the comparator.

## Methods

This randomized, assessor-blinded, positive-controlled, multicentre field trial was conducted in compliance with VICH GL9 (Good Clinical Practice, June 2000) guidelines and Directive 2001/82/EC as amended, applicable European and local and national regulatory requirements (German Drug Law, Hungarian Regulation 128/2009 (X.6.) FVM, Portuguese law N° 148/2008 amended by 413/2009), European Union guidelines for demonstration of efficacy of ectoparasiticides [18] and palatability [19], of veterinary medicinal products; in addition, other applicable guidelines [20, 21].

### Animals

This trial was conducted between March 2015 and August 2015 at 20 veterinary practices in Germany, Hungary and Portugal, in areas with known high prevalence of ticks. Privately owned animals from households with a maximum of three cats and two dogs, were eligible for participation in the trial, provided that the animals did not contact each other or share resting places for the duration of the study. The animal owner was required to provide written consent for his/her animals to be eligible to participate in the trial.

Cats at a minimum age of 2 months, weighing at least 1 kg and classified as clinically healthy and free of any systemic diseases or conditions judged not to interfere with suitability for treatment administration were considered eligible. One cat from each household (primary cat) had to be diagnosed with a tick infestation of at least three live, attached ticks for inclusion in the trial. Any supplementary cats had to fulfil the same eligibility criteria as the primary cats with the exception of tick infestation, i.e. they were included without prior tick count and also in the absence of tick infestation. These animals were not involved in any efficacy assessments and were only monitored for safety and palatability assessments (supplementary cats).

Animals with known hypersensitivity to the active ingredients and/or excipients of the investigational veterinary product (IVP; Credelio^TM^ tablets for cats, active ingredient lotilaner; Elanco Animal Health, Greenfield, USA) or the control product (CP; Eliminall® 50 mg Spot-on solution for cats, active ingredient fipronil; KRKA d.d. Novo mesto, Novo Mesto, Slovenia) were not eligible for the trial. The use of ectoparasiticides with a removal period less than the duration of efficacy mentioned on the label, collars with a withdrawal period of less than 2 weeks, endoparasiticides active against ectoparasites or any products potentially interfering with the trial objectives, was a criterion for exclusion from trial participation. Other criteria for exclusion were pregnant or lactating cats, any animal intended for breeding within 4 months after the last treatment administration, pre-existing medical or surgical conditions that could interfere with the objectives of the study, convalescent animals, and animals scheduled for routine surgical procedures unless they were fully recovered from the procedure. After inclusion in the study, animals could be removed because of a concomitant disease not allowing the animal to stay on the trial or requiring the use of treatments interfering with the trial objectives, death, euthanasia or serious adverse events (AEs). Animals could also be withdrawn prematurely because of protocol non-compliance, use of a non-approved concomitant therapy or owner’s decision.

The animals remained with their owners under their usual housing conditions throughout the trial. Space allocation, thermoregulation, ventilation, and relative humidity were according to the owner’s usual habits or veterinary practices/clinics in case of admission; these parameters were not recorded. Feed and water were according to the owner’s usual habits or according to the veterinary practices/clinics in case of admission. Bathing/immersion in water was to be avoided because of the use of a spot-on treatment as CP.

Except for the IVP or CP administered according to the protocol, the use of any product with efficacy against ticks on the animal or environment between day 0 and the last tick count on day 84 ± 2 was not allowed. Concomitant treatments not interfering with the objectives of the study were allowed. All concomitant treatments were recorded.

### Randomisation and treatment

There were two separate teams (the examining veterinarian’s and the dispenser’s team) at each study site performing the different tasks within the study. The examining veterinarian and laboratory personnel were blinded to the treatment allocation. The sponsor’s representative as well as other study personnel and animal owners were not blinded, but were trained not to provide any treatment information to any blinded study personnel during the study.

Using a block design in a 2:1 ratio (IVP:CP), the primary cats were randomised per household in the sequence of inclusion; all cats (primary and supplementary) in the same household were randomised to the same treatment. Study design, randomisation, definition of inclusion and exclusion criteria, definition of assessment criteria, and description of evaluation criteria and statistical analyses were measures aimed at reducing any bias and provide solid data for analysis.

Treatments (IVP or CP) were administered every 4 weeks on days 0, 28 ± 2 and 56 ± 2. Based on the individual animal’s body weight, animals in Group 1 received the IVP (Credelio^TM^ chewable tablets for cats) at a minimum dose rate of 6.0 mg/kg, orally. Two tablets strengths were available, i.e. 12 mg or 48 mg lotilaner. These two tablets were specifically developed and formulated for cats, with a small tablet size and vanilla-yeast flavouring to aid in administration. The IVP was administered to the cat by the owner under fed conditions, i.e. approximately one-third of the daily ration of regular feed, taken less than 30 min before treatment.

Animals in Group 2 were treated with the CP (Eliminall® 50 mg Spot-on solution): 1 pipette (0.5 ml) topically regardless of body weight, according to the product label. Dogs in the same households (up to two per household) were treated on day 0 or latest on day 1, with an adequate commercial ectoparasiticide provided by the veterinary practice. These dogs had to be under treatment until the end of the study (day 84 ± 2).

### Assessments and analyses

The total duration of the study was between 82 and 86 days per animal except for those prematurely removed from the study. On day 0, all animals were assessed to confirm that they met the eligibility criteria, animal demographics and history were recorded, and cats were allocated to the relevant study group. On days 0, 7 ± 1, 14 ± 2, 21 ± 2, 28 ± 2, 42 ± 2, 56 ± 2, 70 ± 2 and 84 ± 2, the examining veterinarian assessed the primary cats for tick infestation (tick counts), interviewed the owners for any abnormal observation, measured the bodyweight and performed clinical examination (primary and supplementary cats). On day 84 ± 2, all cats were presented for study completion. Cats that were removed from the study early, underwent the same procedure as planned for the study end visit on day 84 ± 2, if possible, and the reason for the animal’s early removal was recorded. The cat owners assessed palatability for all Group 1 cats on days 0, 28 ± 2 and 56 ± 2.

#### Baseline characteristics

Using descriptive statistics, treatment groups were compared for the following baseline parameters: animal characteristics (breed, sex, age, coat length and body weight), animal husbandry. For primary cats, parasite counts on day 0 (live attached ticks and tick species) were also compared. Nominal *P-*values were calculated.

#### Tick counts

The examining veterinarian performed a tick count on each primary cat. The cat was restrained and the entire coat searched, beginning at the head and proceeding systematically to cover all areas of the animal. The hair was pushed manually against its natural lie to expose the skin and attached ticks. If ticks were observed, they were counted, removed, collected, and sent to a laboratory for identification. Before proceeding with the tick removal, the tick viability was assessed. Both live attached and dead attached ticks were counted and collected separately. The examining veterinarian recorded the number of live attached and dead attached ticks.

#### Environmental tick pressure

The environmental tick pressure was assessed and documented as the estimated overall number of animals (cats and dogs) presented at the veterinary practice or clinic, diagnosed with a tick infestation and the number of products supplied for tick prophylaxis in the last 7 days before study enrolment and in the last 7 days before every scheduled follow-up visit of a primary cat.

#### Palatability

Palatability assessment was based on the voluntary acceptance rate defined as the percentage of dosings in which the animals accepted the IVP when offered in an empty bowl or on the ground during 60 s, or when subsequently offered from the hand for an additional 60 s.

#### Endpoints and analysis methods

The primary study endpoint was the average efficacy of the IVP compared to the CP over the entire treatment period compared to baseline, based on counts of live, attached ticks in primary cats. The secondary efficacy criterion was the efficacy of the IVP compared to the CP for each treatment period compared to baseline, again based on live, attached tick counts in primary cats. The experimental unit was the individual animal.

Data from all study animals were entered directly into StudyBase®, a validated CRO-owned Electronic data capture (EDC) system (version 1.7.8.0). All calculations were performed using SAS® statistical analysis software v9.2.2 (SAS Institute Inc., Cary, NC, USA).

Tick counts were log-transformed prior to analysis of covariance (ANCOVA). To avoid taking the log of zero, 1 was added to all tick counts before log-transforming. All statistical differences were assessed at the 2-sided 5% significance level (*P* < 0.05) corresponding to a 1-sided significance level of 2.5%).

The primary analysis of efficacy was based on primary cats in the Per-Protocol (PP) population, but the results were virtually identical for the Intent-to-Treat (ITT) and the PP analysis. Percent efficacy was calculated for the average of all visits and for each visit (days 7 ± 1; 14 ± 2; 21 ± 2; 28 ± 2; 42 ± 2; 56 ± 2; 70 ± 2; and 84 ± 2) as follows:

Percent efficacy = 100 (M0 - MD)/M0

where M0 is the mean tick count on day 0 and MD is the mean tick count on actual day; efficacy was based on live attached ticks.

Tick counts of the IVP treated group were compared to those of the CP group for non-inferiority, with a 20% margin, for each time point during the study and averaged over the whole study period. Non-inferiority was shown if the two-sided 95% confidence interval (CI) for the ratio of tick counts for live attached ticks for the IVP, divided by the same value for the CP, lay within the interval [0, 1/0.80] or [0, 1.25].

All study animals receiving at least one dose of the investigational veterinary or control product were included in the Safety population. This population matched the ITT population. Animals without any major protocol deviation were included in the PP population.

The percentage of adverse events and serious adverse events were compared between the two treatment groups using Fisher’s exact test. Body weight at the various time points were compared between the two treatment groups by ANCOVA with the pre-treatment value as the covariate.

French translation of the Abstract is available in Additional file 1.

## Results

### Animals

A total of 309 cats were randomized in the study: 169 primary cats and 140 supplementary cats. There were 112 and 57 primary cats enrolled in the IVP and CP group, respectively, comprising the ITT population. None of the enrolled primary cats were completely excluded from the PP analysis. Four animals were excluded from the statistical analysis of specific study days; four animals had missing visits and three animals of the efficacy population did not complete the study meeting the criteria for premature removal. Of the 169 primary cats, 47% (*n* = 79), 24% (*n* = 40) and 30% (*n* = 50) were from households with a single cat, 1 supplementary cat and 2 supplementary cats, respectively.

Treatment groups were homogenous for demographic and characteristics at baseline: sex (*P* = 0.6266), age (*P* = 0.7704), body weight (*P* = 0.5854), breed (*P* = 0.9562), hair length (*P* = 0.7208), husbandry (*P* = 1.0000), time spent indoors/outdoors (*P* = 0.1697) and tick counts (*P* = 0.9411) (Table 1).

**Table 1:** Baseline demographics and characteristics (ITT population)

Parameter		Lotilaner (*n* = 112)	Fipronil (*n* = 57)	*Z*-value/ *χ*^2^-value^a^	*P*-value
Age (years)	Arithmetic mean ± SD	4.15 ± 3.38	4.42 ± 3.64	*Z* = 0.29	0.7704
	Minimum–Maximum	0.20–17.0	0.50–14.0		
Sex	Male	60 (54%)	33 (58%)	*Z* = 0.53	0.6266
	Female	52 (46%)	24 (42%)		
Body weight (kg)	Arithmetic mean ± SD	4.1 ± 1.1	4.1 ± 1.0	*Z* = 0.55	0.5854
	Minimum–Maximum	1.5–7.4	1.5–5.8		
Breed	Purebred	21 (19%)	10 (18%)	*χ*^2^_=_ 0.66, *df* = 4	0.9562
	Crossbred	91 (81%)	47 (82%)		
Hair length	Short	81 (72%)	41 (72%)	*Z* = 0.36	0.7208
	Medium	13 (12%)	13 (23%)		
	Long	18 (16%)	3 (5%)		
Husbandry	Countryside	84 (75%)	42 (74%)	*Z* = 0.18	1.0000
	Urban	28 (25%)	15 (26%)		
Time spent	Mostly indoors	19 (17%)	5 (9%)	*Z* = 1.44	0.1697
	Mostly outdoors	93 (83%)	52 (91%)		
Day 0 tick count (live attached)	Number	454	215	*Z* = 0.07	0.9411
	Geometric mean	3.60	3.52	–	–

*Abbreviations*: *n*, number of primary cats; SD, standard deviation

^a^*Z* of a Mann-Whitney test; *χ*^2^ of a Kruskal-Wallis test (of treatment *vs* breed)

Following enrolment, 7 cats (3 in the IVP group and 4 in the CP group) were removed from the study because of the following reasons: death (3; 2 cats were killed in car accidents and 1 cat died because of respiratory failure secondary to acute-subacute aspiration pneumonia and pleural effusion), pregnancy (1), owner’s non-compliance (2) and loss to follow-up (1).

### Efficacy

At each administration day, all study animals received the correct amount of IVP or CP as defined in the protocol. The efficacy analysis population (both PP and ITT) included 169 primary cats with 112 and 57 cats in the IVP and CP groups, respectively. Because no cats were completely excluded from the PP analysis and 4 cats were only partially excluded (2 each in the IVP and CP groups), the results of the efficacy analysis for the ITT and PP populations are almost identical. Results are presented here for the PP population. The geometric mean tick count at baseline was 3.60 and 3.52 in the IVP and CP groups, respectively.

It was demonstrated (with a 97.5% confidence limit) that tick counts in the IVP group were not higher than those in the CP group, up to a non-inferiority margin of 20% over all visits. Superiority of lotilaner compared to fipronil was demonstrated on the majority of study days [day 14 to day 70, when cats treated with lotilaner had significantly lower tick counts (*P* < 0.0394)], as well as for efficacy averaged over all time points (*P* < 0.0001) (Table 2).

**Table 2:** ANCOVA analysis for non-inferiority testing - live attached tick counts (PP)

Day	Lotilaner (*n* = 112)			Fipronil (*n* = 57)			Comparison		95% CI
	Geometric mean tick count	Est	SE	Geometric mean tick count	Est	SE	*t*-value	*P*-value^a^	
0	3.60	na	na	3.52	na	na	–	–	–
7	0.00	0.00	0.01	0.01	0.01	0.01	*t*_(162)_ = 1.32	0.1876	0.97–1.01
14	0.00	0.00	0.01	0.04	0.05	0.02	*t*_(164)_ = 2.08	**0.0394**	0.92–1.00
21	0.00	0.01	0.02	0.09	0.10	0.03	*t*_(164)_ = 2.82	**0.0054**	086–0.97
28	0.06	0.06	0.04	0.37	0.35	0.07	*t*_(162)_ = 4.04	**< 0.0001**	0.69–0.88
42	0.00	0.00	0.03	0.22	0.22	0.04	*t*_(163)_ = 4.53	**< 0.0001**	0.75–0.89
56	0.04	0.03	0.03	0.22	0.21	0.05	*t*_(162)_ = 3.25	**0.0014**	0.78–0.94
70	0.01	0.01	0.02	0.11	0.10	0.03	*t*_(160)_ = 2.42	**0.0167**	0.85–0.98
84	0.00	0.00	0.01	0.01	0.01	0.01	*t*_(159)_ = 1.36	0.1747	0.97–1.01
Mean	0.01	0.02	0.02	0.13	0.18	0.03	*t*_(164)_ = 5.16	**< 0.0001**	0.82–0.91

*Abbreviations*: Est, estimate; na, not applicable; SE, standard error

^a^Significant values (*P* < 0.05) in bold

**Fig. 1:**
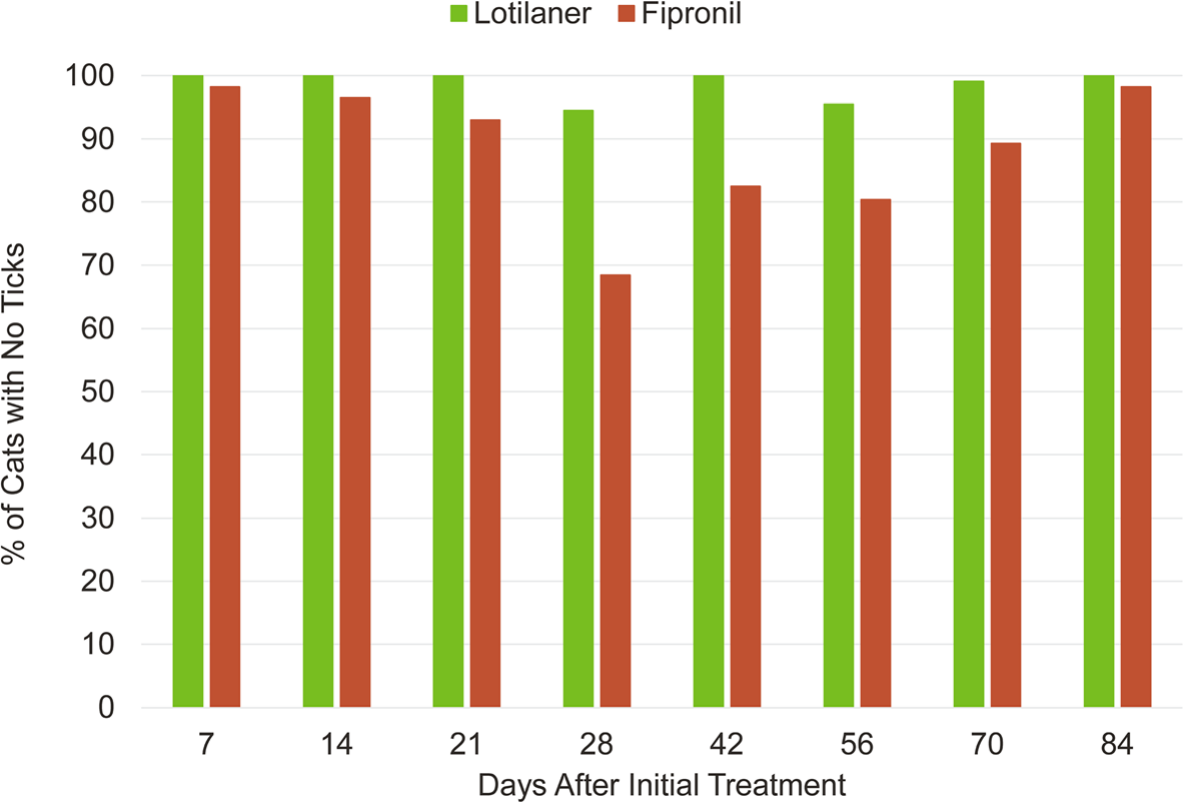
Percentage of cats free of live ticks after treatment with lotilaner or fipronil throughout the study

Cure rate was defined as percentage of cats with zero live attached ticks. Cure rates in the IVP group were 100%, 100%, 100%, 94.5%, 100%, 95.5%, 99.1% and 100% on days 7, 14, 21, 28, 42, 56, 70 and 84, respectively. The corresponding cure rates in the CP group were 98.2%, 96.5%, 93%, 68.4%, 82.5%, 80.4%, 89.3% and 98.2%, respectively (Fig. 1).

The baseline data for tick species and numbers are provided in Table 3. The study period covered the normal period of activity for the tick species *Ixodes ricinus*, *Rhipicephalus sanguineus*, *Dermacentor reticulatus* and *Ixodes hexagonus*, and persistent challenge was demonstrated. The overall mean percentage efficacy, as well as the efficacy against the various identified tick species in this study for the IVP and CP treated groups over the treatment period compared with baseline, are shown in Table 4 and Table 5.

**Table 3:** Tick species and numbers at baseline (ITT)

Ticks		Lotilaner			Fipronil			Comparison (live ticks)	
		No. of subjects	Live ticks	Dead ticks	No. of subjects	Live ticks	Dead ticks	*Z*-value	*P*-value
All		112	454	1	57	215	2	0.07	0.9411
Genus	*Rhipicephalus*	32	132	0	14	50	0.50	0.50	0.6175
	*Dermacentor*	26	58	0	15	39	0.60	0.60	0.5515
	*Ixodes*	75	264	1	38	126	0.19	0.19	0.8480
Species	*Rhipicephalus sanguineus*	32	132	0	14	50	0.50	0.50	0.6175
	*Dermacentor reticulatus*	26	58	0	15	39	0.60	0.60	0.5515
	*Ixodes ricinus*	69	249	1	35	113	0.39	0.39	0.6989
	*Ixodes hexagonus*	6	15	0	6	13	1.21	1.21	0.2277
Morphotype	*Rhipicephalus sanguineus*, morphotype 2	31	103	0	14	40	0.53	0.53	0.5986
	*Rhipicephalus sanguineus*, morphotype unknown	10	29	0	8	10	0.99	0.99	0.3246
Stage	Adult	110	444	1	55	202	0.49	0.49	0.6222
	Nymph	4	7	0	5	10	1.43	1.43	0.1535
	Larva	1	3	0	1	3	0.48	0.48	1.0000
Sex	Male	69	126	0	32	52	0.92	0.92	0.3591
	Female	107	318	1	54	150	0.11	0.11	0.9120
	Not determinable	5	10	0	6	13	1.51	1.51	0.1304

**Table 4:** Mean percentage efficacy against ticks compared with baseline (PP)

Day	Lotilaner		Fipronil	
	Geometric mean tick count	Efficacy (%)	Geometric mean tick count	Efficacy (%)
0	3.60	na	3.52	na
7	0	100	0.01	99.6
14	0	100	0.04	98.8
21	0	100	0.09	97.5
28	0.06	98.3	0.37	89.6
42	0	100	0.22	93.7
56	0.04	98.9	0.22	93.6
70	0.01	99.6	0.11	96.9
84	0	100	0.01	99.6
Mean	0.01	99.6	0.13	96.4

*Abbreviation*: na, not applicable

**Table 5:** Mean percentage efficacy against tick species over the treatment period compared with baseline (PP)

Species	Time point	Lotilaner		Fipronil	
		Geometric mean tick count	Geometric % efficacy	Geometric mean tick count	Geometric % efficacy
*Ixodes ricinus*	Day 0	1.36	99.0	1.22	91.7
	Mean	0.01		0.10	
*Rhipicephalus sanguineus*	Day 0	0.53	100	0.45	97.2
	Mean	0		0.01	
*Dermacentor reticulatus*	Day 0	0.29	99.6	0.38	100
	Mean	0		0.00	
*Ixodes hexagonus*	Day 0	0.07	100	0.12	89.6
	Mean	0		0.01	

### Palatability

Palatability was assessed for all primary and supplementary cats in the IVP group. Administration compliance in the lotilaner group was 100%, with all cats successfully dosed by their owners and no refusal of tablets recorded. The mean voluntary acceptance rate (pooled data from all 3 monthly treatment days) was 48%.

### Safety

The safety population included all primary and supplementary animals. In total, 309 cats were included in the safety population with 211 and 98 in the IVP and the CP group, respectively.

#### Adverse events

Over the study duration, 18 adverse events [AEs and serious adverse events (SAEs)] were reported in 7 of the 309 animals with 3 of 211 cats (1.0%) in the IVP group and 4 out of 98 cats (1.3%) in the CP group showing at least 1 clinical sign.

With the exception of 2 deaths in the Eliminall® group (frequency = 2%), all other AEs were reported in only one cat each, with a corresponding frequency of 0.5% in the Credelio^TM^ group and 1% in the Eliminall® group. No vomiting nor diarrhoea were observed in the study, in any of the treatment groups. Using Fisher’s exact test, no statistical significant difference was found between the 2 treatment groups for any of the reported AEs or SAEs (*P* = 0.2138 and *P* = 0.2372, respectively).

Three cats had SAEs during the study, none of which were attributed to either treatments. Two cats in the CP group died because of car accidents. One 12-year-old cat in the IVP group was brought to the clinic on study day 22 due to complaints of the upper respiratory tract and corneal injury and was administered vitamins, a subcutaneous infusion and an oral endoparasiticide containing milbemycin oxime and praziquantel; a first dose of meloxicam suspension and of marbocyl tablets were also given by the examining veterinarian and continued by the animal owner at home. On day 26, the cat was presented to the clinic with severe dyspnoea, tachycardia, cyanosis and dehydration and in spite of all efforts, died after 40 min because of respiratory failure. Necropsy revealed lung necrosis and pleural effusion and the cat’s symptoms were attributed to acute-subacute aspiration pneumonia with consequential respiratory failure and shock. All AEs and sAEs were considered unrelated to the IVP administration.

#### Body weight

The mean (SD) baseline body weight was 4.10 (1.00) kg and 3.90 (1.00) kg for cats in the IVP and CP groups, respectively. Group comparisons of body weight using RMANCOVA showed no significant difference between the treatment groups at any of the time points (*P* = 0.9739).

### Environmental tick pressure

**Fig. 2:**
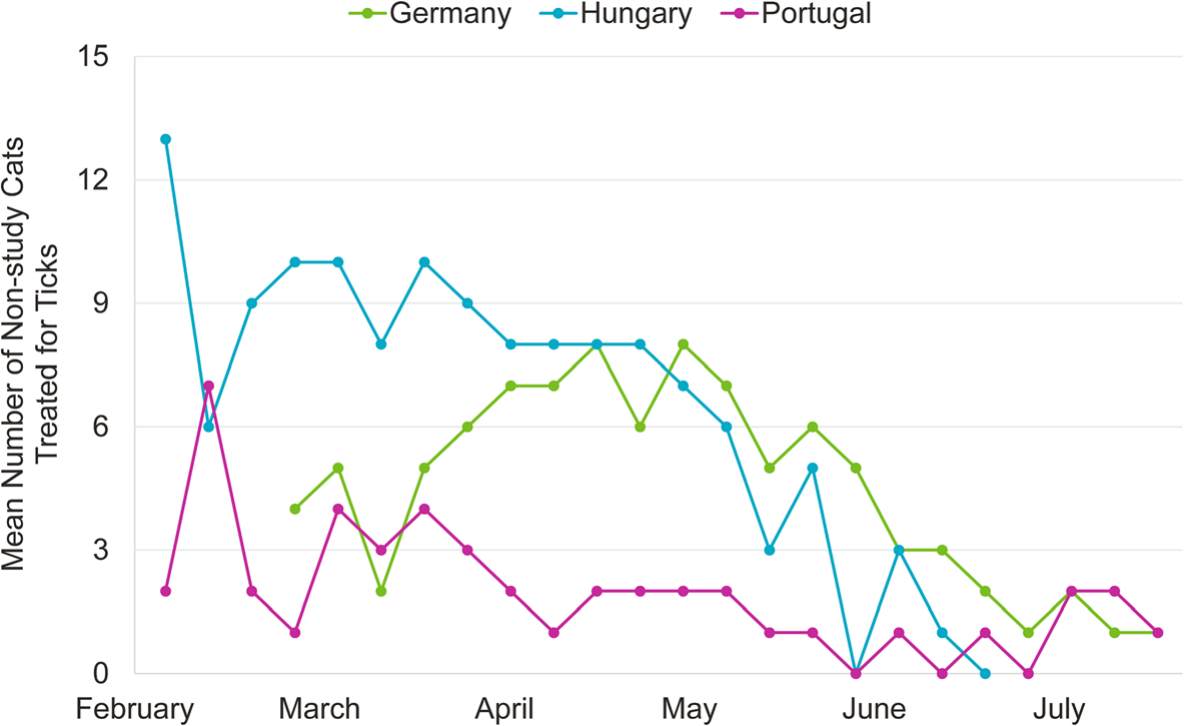
Mean number of non-study cats treated for tick infestations; average over study sites within countries at weekly intervals throughout the study

The number of animals diagnosed with tick infestation during the 7 days prior to a study visit ranged between 0–13 cats and 0–18 dogs. The number of individual products dispensed for tick prophylaxis during the 7 days prior to a study visit ranged between 1–20 cats and 1–74 dogs (Fig. 2).

## Discussion

This field study compared the safety and the efficacy of the lotilaner commercial formulation (Credelio^TM^) against ticks when administered orally at the minimum dose rate of 6 mg/kg, *vs* a fipronil spot-on (Eliminall®, in cats. The choice of the three different regions in which the study was performed ensured the evaluation of the efficacy of the product in different climatic and geographical conditions, with a high environmental tick infestation pressure.

The comparison between an orally-administered product (lotilaner) against a spot-on treatment (fipronil) was driven by the lack of availability of an oral tick product for cats. Furthermore, a generic fipronil containing product was preferred over the more commonly used Frontline® spot-on for cats, because of its consistent 4 weeks efficacy duration against *I. ricinus* [22], as opposed to the shorter duration claimed by Frontline®, as short as 2 weeks, depending on the country, [23].

The minimum body weight of the cats for inclusion (1 kg) was driven by the comparator product. In pilot and pivotal target animal safety studies (unpublished data and 22, respectively), lotilaner was shown to be safe for cats as light as 0.5 kg, but since Eliminall® was contraindicated for cats lighter than 1 kg, a minimum bodyweight of 1 kg at inclusion was selected in order to maintain the blinding and prevent the introduction of a bias.

Tick counts and analysis of the demographic parameters and related variables (age, sex, body weight, breed, hair length, living area: countryside *vs* urban, and lifestyle, i.e. time spent primarily indoors *vs* outdoors) confirmed that the two treatment groups were homogeneous at baseline, with no statistically significant difference in any of the variables.

In the lotilaner group the efficacy *vs* baseline was above 98% at all time points and 99.6% averaged over all time points. In the fipronil group the efficacy was above 89% at all time points and 96.4% averaged over all time points.

Lotilaner was proven to be superior to fipronil on the majority of study days, i.e. from day 14 to day 70, when cats treated with lotilaner had significantly lower tick counts (*P* < 0.0394), and was non-inferior to the control product on the other assessment days. Superiority of lotilaner was shown as well for efficacy averaged over all time points (*P* < 0.0001).

Although in the pivotal laboratory studies the efficacy of lotilaner was only tested against the most common European cat tick (*Ixodes ricinus*) [17], the results of this field study demonstrate that the product is equally efficacious against the other clinically relevant tick species infesting cats in Europe, i.e. *Rhipicephalus sanguineus*, *Dermacentor reticulatus* and possibly *I. hexagonus*, although the low number of these ticks at baseline does not allow for a firm conclusion on efficacy. The species specific analysis showed an efficacy of 100% against *R. sanguineus* and *I. hexagonus* at all time points and an average efficacy of 99.6% against *D. reticulatus*.

Credelio^TM^ chewable tablets for cats was designed specifically as a small, vanilla flavoured, chewable tablet, to ensure ease of administration and acceptance by cats; 100% compliance in the lotilaner group confirmed that the tablets were easy for pet owners to administer and well accepted by cats. Palatability results showed that the product had been taken voluntarily by 48% cats, which is in line with the “pickiness” and well documented individual eating preferences of cats [24], as well as their aversion to unfamiliar flavours and textures [25].

Both treatments were well tolerated, with 0.014% lotilaner cats and 0.4% cats treated with fipronil affected by adverse events. The difference was not statistically significant. In addition, no significant differences between the two groups in body weight change were observed.

## Conclusions

Lotilaner tablets for cats, used at a minimum dose rate of 6.0 mg/kg, were shown to be effective and safe for the treatment of tick infestations in cats presented as patients to veterinary practices under field conditions in Germany, Hungary and Portugal. Persistent efficacy lasted for one month and lotilaner was superior to fipronil for efficacy averaged over all time points (*P* < 0.0001) as well as on most individual assessment days (day 14 to 70, *P* < 0.0394), and was non-inferior to fipronil on the other days. Lotilaner tablets were easy for pet owners to administer and well accepted by cats.

## Additional file

Additional file 1: French translation of the Abstract. (PDF 18 kb)

## Abbreviations

AE: Adverse event
ANCOVA: Analysis of covariance
CI: Confidence interval
CP: Control product
EDC: Electronic data capture
ITT: Intent-to-treat
IVP: Investigational veterinary product
PP: Per protocol
SAE: Serious adverse event
SD: Standard deviation
