# Supplementary material for: A randomized, controlled field study to assess the efficacy and safety of lotilaner (Credelio™) in controlling ticks in client-owned cats in Europe
Source: Parasit Vectors. 2018 Jul 13;11:411. doi: 10.1186/s13071-018-2967-5 (PMC6043961; doi:10.1186/s13071-018-2967-5)
Supplement: Supplementary file 1 — French translation of the Abstract. (PDF 18 kb) [file 13071_2018_2967_MOESM1_ESM.pdf]

Étude de terrain contrôlée et randomisée visant à évaluer l'efficacité et l'innocuité du lotilaner (Credelio™) pour le contrôle des tiques sur des chats de compagnie en Europe

Daniela Cavalleri[1*], Martin Murphy[1], Wolfgang Seewald[1] et Steve Nanchen[1]

[1]Elanco Animal Health, Mattenstrasse 24a, CH-4058, Bâle, Suisse.

*Correspondance : cavalleri_daniela_a@elanco.com

Adresses électroniques :
Daniela Cavalleri : cavalleri_daniela_a@elanco.com
Martin Murphy : murphy_martin_gerard@elanco.com
Wolfgang Seewald : seewald_wolfgang@elanco.com
Steve Nanchen : nanchen_steve@elanco.com

Résumé

Contexte : Chez le chat, le contrôle et le traitement de l'infestation par des tiques constituent un enjeu permanent qui nécessite sans cesse de nouvelles approches. Le lotilaner, un agent à action rapide de la famille des isoxazolines, a prouvé son efficacité contre les tiques dans le cadre d'études de laboratoire. Une étude a été menée sur des chats de compagnie afin de confirmer l'efficacité et l'innocuité du lotilaner, à la dose minimale de 6,0 mg/kg, contre les espèces de tiques infestant le chat les plus fréquemment rencontrées en Europe.

Méthodes : Vingt cliniques situées en Allemagne, en Hongrie et au Portugal ont participé à cette étude. Les foyers sélectionnés, ne comptant pas plus de trois chats, ont été répartis aléatoirement selon un rapport de 2/1 entre les deux groupes de traitement, à savoir : lotilaner ou fipronil. Dans chaque foyer, le premier chat hébergeant au moins trois tiques fixées et vivantes a été sélectionné comme chat principal. Les traitements ont été délivrés aux propriétaires qui devaient les administrer à J0, J28 et J56. Un comptage des tiques a été réalisé à J0, J7, J14, J21, J28, J42, J56, J70 et J84 sur les chats principaux ; les autres chats ont uniquement fait l'objet d'une évaluation de l'innocuité à J28, J56 et J84. L'efficacité était évaluée en comparant le nombre de tiques fixées et vivantes à J0 à celui des comptages suivants.

Résultats : Les espèces de tiques les plus fréquemment extraites étaient *Ixodes ricinus*, *Rhipicephalus sanguineus* et *Dermacentor reticulatus*, l'espèce *Ixodes hexagonus* ayant également été isolée. Dans le groupe lotilaner (n = 112), l'efficacité (évaluée à partir de la moyenne géométrique du nombre de tiques) se situait entre 98,3 et 100 %. Celle du fipronil (n = 57) était comprise entre 89,6 et 99,6 %, avec des tiques vivantes et fixées constatées sur certains chats à chaque comptage. À J21, J28, J42 et J56 (P < 0,05), le nombre moyen de tiques chez les chats traités au lotilaner était significativement inférieur à celui des chats traités au fipronil. L'efficacité moyenne (en pourcentage) mesurée pour toutes les visites suivant l'inclusion était de 99,6 % et 96,4 %, dans les groupes lotilaner et fipronil, respectivement (P < 0,0001). Le lotilaner s'est avéré supérieur au fipronil en termes d'efficacité moyenne établie sur l'ensemble des comptages (p < 0,0001) et pour certains jours d'évaluation individuelle (J14 à J70, P < 0,0394) ; il s'est montré non inférieur au fipronil pour les autres jours. Les propriétaires ont réussi à administrer tous les traitements et les deux produits ont été bien tolérés.

Conclusions : Credelio™ s'est avéré efficace et sans danger dans le traitement des infestations par les tiques chez le chat de compagnie. Son efficacité a duré un mois et le lotilaner s'est montré supérieur au fipronil lors de la plupart des évaluations. Les taux de succès du traitement étaient compris entre 94,5 et 100 % pour le lotilaner et 68,4 et 98,2 % pour le fipronil.

Mots clés : Credelio, lotilaner, fipronil, chat, tique, efficacité, étude de terrain, innocuité, Europe.
